# Polaritonic and excitonic semiclassical time crystals based on TMDC strips in an external periodic potential

**DOI:** 10.1038/s41598-023-46077-0

**Published:** 2023-11-11

**Authors:** Gabriel P. Martins, Oleg L. Berman, Godfrey Gumbs

**Affiliations:** 1 Physics Department, New York City College of Technology, The City University of New York, 300 Jay Street, Brooklyn, NY 11201 USA; 2 The Graduate School and University Center, The City University of New York, 365 Fifth Avenue, New York, NY 10016 USA; 3 Department of Physics and Astronomy, Hunter College of the City University of New York, 695 Park Avenue, New York, NY 10065 USA; 4 Donostia International Physics Center (DIPC), P de Manuel Lardizabal, 4, 20018 San Sebastian, Basque Country Spain

**Keywords:** Bose-Einstein condensates, Surfaces, interfaces and thin films

## Abstract

We investigated the dynamics of Bose–Einstein condensates (BECs) under an external periodic potential. We consider two such systems, the first being made of exciton–polaritons in a nanoribbon of transition metal dichalcogenides (TMDCs), such as MoSe$_2$, embedded in a microcavity with a spatial curvature, which serves as the source of the external periodic potential. The second, made of bare excitons in a nanoribbon of twisted TMDC bilayer, which naturally creates a periodic Moiré potential that can be controlled by the twist angle. We proved that such systems behave as semiclassical time crystals (TCs). This was demonstrated by the fact that the calculated BEC spatial density profile shows a non-trivial long-range two-point correlator that oscillates in time. These BECs density profiles were calculated by solving the quantum Lindblad master equations for the density matrix within the mean-field approximation. We then go beyond the usual mean-field approach by adding a stochastic term to the master equation which corresponds to quantum corrections. We show that the TC phase is still present.

## Introduction

Symmetry breaking has been a subject of much interest in condensed matter physics since it leads to further understanding of many phases of matter exhibited by materials. In symmetry-broken phases, a system has a Hamiltonian which is unchanged by a particular symmetry transformation, but the state of the system itself does not exhibit the same symmetry. The breaking of translational symmetry, for example, leads to a crystal phase; the breaking of spin-rotational symmetry gives rise to a ferromagnetic phase. A Time crystal (TC) is a state of matter which shows spontaneous breaking of time translation symmetry (TTS). This phase was first predicted by Wilczek in 2012^1^ and has been the subject of many studies ever since. In order for a system to be considered a TC, it has to contain a macroscopically large number of particles which exhibit long-living spontaneous breaking of TTS, showing non-trivial correlations in an order parameter when measured at two long-time apart instances^2^. A proper mathematical definition for a Quantum TC was proposed in Ref.^3^.

Many no-go theorems have been advanced showing that TCs cannot exist under certain conditions^3–5^. Ever since then, several groups have proposed different settings that could exhibit a TC phase^6–9^. In a recent review, Sondhi et al. presented a detailed and very well written description of TCs^2^. Systems ranging from chains of spins^10,11^ to Bose–Einstein Condensates (BECs) in many different settings^12–16^ have been shown to display spontaneous breaking of the TTS. Of particular interest to our work is the one found in Ref.^13^, in which a BEC of exciton-polaritons in an annular potential in which a TC phase was shown to be present within the annular region.

Technological applications of time crystals are the subject of current studies. Many TC settings have been demonstrated to be very resilient to external perturbations^17–19^. It has been shown that coupling TCs to entangled systems prevents the otherwise very fragile quantum entanglement from rapid decay due to the interactions of these entangled systems with the external environment^17^. Therefore, it has been proposed that TCs are very appropriate candidates to be applied for the development of memory-storing apparatus for quantum computers^20^. Additionally, in recent times it has been discussed that a set of TCs can be employed as a model to simulate the human brain^21^.

Absorption of a photon by a semiconductor leads to the creation of an electron in the conduction band and a positive charge, i.e., “hole”, in the valence band. This electron–hole pair can form a bound state referred to as an “exciton”^22^. Bose–Einstein condensation and superfluidity of such excitons are expected to exist at experimentally observed exciton densities at temperatures much higher than for the BEC of alkali atoms^22,23^. A direct exciton is a two-dimensional (2D) exciton, formed as a bound state by an electron and a hole in a single semiconductor quantum well whereas an indirect exciton is formed by the bound state of an electron and a hole in neighboring quantum wells. Excitons can be created when the material absorbs photons and can decay by emitting photons. When a suitable material for the occurrence of excitons is put inside an optical microcavity, linear superposition between photons and excitons can be found^24^. Such a quasiparticle is known as an exciton–polariton.

Many theoretical and experimental investigations have identified Bose coherent effects of 2D excitonic polaritons in a quantum well embedded in a semiconductor microcavity^24–27^. To obtain polaritons, two Bragg mirrors are placed opposite each other in order to form a microcavity, and a quantum well is embedded within the cavity at the antinodes of the confined optical mode. The resonant interaction between a direct exciton in a quantum well and a microcavity photon results in the Rabi splitting of the excitation spectrum. Two polariton branches appear in the spectrum due to the resonant exciton–photon coupling. The lower polariton branch of the spectrum has a minimum at zero momentum. These lower polaritons form a 2D weakly interacting Bose gas. The extremely light mass of these bosonic quasiparticles at experimentally achievable excitonic densities results in a relatively high critical temperature for superfluidity. The critical temperature is relatively high because the 2D thermal de Broglie wavelength is inversely proportional to the mass of the quasiparticle, and this wavelength becomes comparable to the separation between the bosons. BEC and superfluidity of exciton-polaritons have been observed in a microcavity^26–28^. The various applications of microcavity polaritons for optoelectronics and nanophotonics have been developed recently^24^.

Two-dimensional van der Waals materials such as the atomically thin transition metal dichalcogenides have unique physical properties, which are attractive for a broad range of applications. Monolayers of TMDC such as ${\textrm{MoS}}_{2}$, ${\textrm{MoSe}}_{2}$, ${\textrm{MoTe}}_{2}$, ${\textrm{WS}}_{2}$, ${\textrm{WSe}}_{2}$, and ${\textrm{WTe}}_{2}$, for instance, are 2D direct bandgap semiconductors, which have a variety of applications in electronics and optoelectronics^29^. The strong interest in TMDC monolayers is driven by the following factors: the direct gap in the band structure spectrum^30^, the existence of excitonic valley physics, and the possibility of electrically tunable, strong light–matter interactions^31,32^. Monolayer TMDCs have already been implemented in field-effect transistors, logic devices, and lateral and tunneling optoelectronic structures^29^. Monolayer TMDCs have hexagonal lattice structures, and the nodes (valleys) in the dispersion relations of the valence (conduction) band can be found at the $\textbf{K}$ and $\textbf{K}^{\prime }$ points of the hexagonal Brillouin zone. The specific properties of excitons in monolayer TMDCs have been the subject of many experimental and theoretical studies (see, for example, Ref.^33^). The large binding energy and long lifetime of interlayer excitons in van der Waals heterostructures have prompted much work on these materials^34,35^. Exciton–polaritons in a TMDC monolayer embedded in a microcavity were observed experimentally at room temperature^36^. The superfluidity of exciton–polaritons in a TMDC monolayer embedded in a microcavity has been studied in Refs.^37–39^.

Stacking 2D materials to form van der Waals heterostructures opens up new strategies for materials properties engineering. One increasingly important example is the possibility of employing the relative orientation (twist) angle between a pair of 2D crystals to tune electronic properties. For small twist angles and lattice constant mismatchings, heterostructures exhibit long period Moiré patterns, characterized by the periodic potential acting on the charge carriers^40^. It is well established that the Moiré superlattice can modulate the electronic band structure of the material and lead to transport properties such as unconventional superconductivity^41^ and insulating behavior driven by correlations^42–44^.

In bilayer TMDC, formed by vertically stacking two TMDC monolayers, intralayer excitons are formed by an electron and a hole, located in the same monolayer. On the other hand, interlayer excitons are formed by an electron and a hole, located in two neighboring monolayers. Since an interlayer exciton is composed of an electron and a hole which are separated in neighboring layers, its properties can depend strongly on the layer configurations and external fields. For example, it was recently predicted that Moiré superlattices, where the interlayer atomic registry changes periodically over space, can host arrays of localized interlayer exciton states with distinct valley selection rules^45–47^.

In this paper, we investigate the dynamics of two BEC systems. One made of exciton-polaritons in a strip of a MoSe$_2$ monolayer, embedded in a microcavity, and the other made of bare excitons on a strip of twisted TMDC bilayer. We show that, under certain circumstances, these systems are good candidates for time crystallization. In the dilute regime, the systems will form a BEC whose mean-field dynamics is given by Gross–Pitaevskii’s equation (GPE). For the exciton-polaritons, we consider the strip to be inside a spatially curved optical microcavity. The curvature of the cavity creates an effective external potential on the photons inside the cavity which, in turn, acts as an effective potential for the polariton as a whole^37–39^. This effective potential enters directly in the GPE and significantly changes the dynamics of the condensate. We calculate the curvature required for an effective sinusoidal potential on the polariton BEC. For the bare excitons system, the Moiré pattern caused by the relative twist between the two TMDC layers naturally creates a periodic external potential for the excitons in the strip. We present the mathematical condition for a system to be in a semiclassical TC phase. This condition is a modification of the quantum TC criterion proposed in Ref.^3^. We numerically solved the GPE which governs the time evolution of both considered systems within the mean-field approach and showed that both systems obey the semiclassical TC criterion. We also go beyond the mean-field description by adding a stochastic term to the GPE^27^ and show both systems still demonstrate the semiclassical TC behavior.

A study implying that a BEC of exciton-polaritons can behave as a time crystal has been reported in Ref.^13^. However, there are some considerable differences between the system studied in Ref.^13^ and the one under consideration in our paper. While in Ref.^13^ a localized TC based on exciton-polaritons inside a 2D annular trap was analyzed, we consider an extended system in a very long strip within an external periodic potential. The dynamics in both systems are significantly different, even though both of them follow similar GPEs. Besides, we also have considered our systems beyond the mean field approach.

The rest of this paper is organized as follows. We present the theoretical foundation of the problem in “Theoretical framework” section, where we define both systems which we studied. We also present the mathematical framework for semiclassical time crystals. In “Methods” section, we present model equations for the dynamics of a non-equilibrium BEC in a periodic potential, both in the usual mean-field description, as well as showing how to go beyond that, by adding a stochastic term corresponding to quantum uncertainty in the equations. Numerical results of our calculations for the mean-field dynamics of an exciton–polariton and bare-exciton condensate in a periodic potential are presented in “Numerical results” section.

## Theoretical framework

In this section, we provide the theoretical framework for the remainder of the paper. First, we define our systems of interest, namely an exciton–polariton condensate on a strip of TMDC inside a spatially curved microcavity and an excitonic BEC on a strip of twisted TMDC bilayer. After that, we provide the mathematical condition that must be satisfied by semiclassical time crystals.

### Exciton–polariton BEC in an uneven microcavity

In semiconductors, electrons in the conduction band and holes in the valence band can be bound together, forming a hydrogen-like structure called an exciton^22^. The creation energy for the excitons $\varepsilon _{exc}$ is equal to the gap energy between the conduction and valence bands of the semiconductor, $\Delta$, minus the binding energy of the electron-hole pair $\varepsilon _b$, $\varepsilon _{exc} = \Delta -\varepsilon _{b}$. Excitons are created when the material absorbs photons and can spontaneously decay by the recombination of the electron-hole pair, which, in turn, emits a photon. When a material that can harbor excitons is contained within an optical microcavity, in which photons are confined, a linear superposition state can be formed between excitons and photons. Those states are called exciton–polaritons^24^. There are two branches of exciton–polaritons, one with higher energy, called the *upper polariton* branch, and one with lower energy, called the *lower polariton* branch. The lower polaritons can form Bose–Einstein condensates^24^. One such condensate has been recently verified in room-temperature settings^28^.

**Figure 1:**
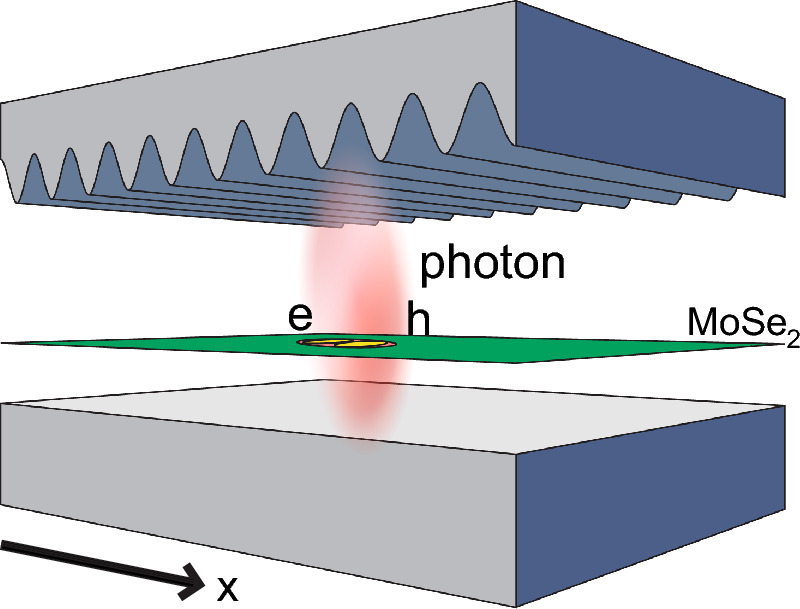
Schematic representation of the considered system. A strip of MoSe$_2$ inside an uneven microcavity. The cavity is composed of a plane mirror at the bottom and a spatially curved mirror on the top. The cavity length is, therefore, not constant and is a function of *x*, $L_C(x)$, given by Eq. (1).

Inside an optical microcavity of length $L_C$, the energy of a photon of the *q*-th mode with momentum $\textbf{P}$ is $\varepsilon _{ph}(\textbf{P}) = (c/n)\sqrt{P^2+\hbar ^2 \pi ^2 q^2 {L_C}^{-2}}$, where $n = \sqrt{\epsilon _r}$ is the refractive index of the cavity. For low momentum, $\varepsilon _{ph} \approx \dfrac{\hbar \pi q c}{n L_C}$. If this cavity is uneven, namely, if one of the mirrors that make it is curved, the energies of the photons depend on the position. This effectively creates an external potential, $V_{\textrm{ph}}$, for low-momentum photons inside the cavity. If, for example, a cavity has a length that varies in the *x* direction like

1 $$\begin{aligned} L_C (x) = \frac{\hbar \pi q c}{n\left( \varepsilon _{0}+V_{\textrm{ph}}(x)\right) }, \end{aligned}$$

low-momentum photons of the *q*-th mode will have an energy $\varepsilon _{\textrm{ph}}(x) = \varepsilon _0 +V_{\textrm{ph}}(x)$. It is evident that the effect of such a curvature is equivalent to the addition of an external potential to the photons. It can be shown that an effective potential on either photons, $V_{\textrm{ph}}(x)$, or excitons, $V_{\textrm{exc}}(x)$, leads to an effective potential $V_{\textrm{eff}}$ acting on exciton-polaritons, which can be approximated as $V_{\textrm{eff}}=\dfrac{1}{2}\left( V_{\textrm{ph}}+V_{\textrm{exc}}\right)$^37–39^.

We consider the system depicted in Fig. 1. Such a system is composed of exciton-polaritons in a strip of TMDC embedded in a curved optical microcavity with the length $L_C(x)$ given by

2 $$\begin{aligned} L_C (x) = \frac{\hbar \pi q c}{n\left( \varepsilon _{\textrm{exc}}+2V_0 \cos (kx)\right) } . \end{aligned}$$

Polaritons confined in such a cavity would be subjected to an effective potential $V_{\textrm{eff}}(x) = V_0\cos (kx)$, $k = 2\pi /a_{p}$ with $a_{p}$ being a period of the external effective potential, and $V_0$ the amplitude, which can both be, in principle, arbitrarily chosen by the design of the microcavity.

### Excitonic BEC in a twisted TMDC bilayer

**Figure 2:**
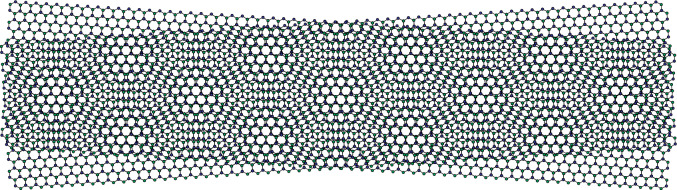
Moiré pattern in the crystal lattice structure seen by twisting one of the layers of a bilayer TMDC. The pattern is created by the difference in atomic alignment in the upper and lower layers. Excitons in such an environment will be subject to an external periodic potential given by Eq. (3).

We also consider a system composed of bare excitons in a twisted TMDC bilayer as shown in Fig. 2. Bare excitons can also form a BEC phase at low temperatures^22,23^.

It has been shown that, when excitons are in a twisted TMDC bilayer, they are subjected to an effective periodic potential $V(\textbf{r})$ caused by the Moiré pattern given by^40^

3 $$\begin{aligned} V(\textbf{r})= & {} V_0\sum _j \cos \left( k\textbf{b}_j\cdot \textbf{r}\right) \nonumber \\= & {} 2V_0 \left( \cos (kx) + \cos k\left( \frac{x}{2}+\dfrac{\sqrt{3}}{2}y\right) +\cos k\left( \frac{x}{2}-\dfrac{\sqrt{3}}{2}y\right) \right) , \end{aligned}$$

where $\textbf{b}_j$ are unit vectors which divide the plane into six identical sections, and $k=\frac{2\pi }{a_M}$, where $a_M$ is the Moiré period. If we constrain the excitons to a ribbon of TMDC that is finite in the $\textit{y}$ direction with a width *W* and infinitely long in the $\textit{x}$ direction, we can replace the potential by an effective potential $V_{\textrm{eff}}(x)$ given by

4 $$\begin{aligned} V_{\textrm{eff}}(x) = \frac{1}{W}\int _{-W/2}^{W/2}dy\ V(x,y) = 2V_0 \cos (kx), \end{aligned}$$

only if the width $W = \dfrac{4\pi m}{k\sqrt{3}}=\dfrac{2m}{\sqrt{3}}a_M,$ for positive integer *m*’s.

### Criterion for a semiclassical time crystal

The mathematical criterion that a system must obey in order for it to be a quantum time crystal was first developed in Ref.^3^. This criterion, however, is not appropriate for the study of mean-field dynamics, which is the subject of studies in this paper. Therefore, we needed to devise a new criterion that can be applied to the semiclassical mean-field study of BECs.

By following steps similar to the ones in Ref.^3^, we say that a system behaves as a semiclassical time crystal if it shows non-trivial time dependent correlations between values of an order parameter $\rho (\textbf{r},t)$ at long-apart times and positions. Namely, if

5 $$\begin{aligned} \lim _{|\Delta \textbf{r}| \rightarrow \infty } \lim _{|t-t^\prime |\rightarrow \infty }\dfrac{1}{V}\int \rho (\textbf{r},t) \rho (\textbf{r}+\Delta \textbf{r} ,t^\prime ) d\textbf{r} = c(t), \end{aligned}$$

where *c*(*t*) is a non-stationary function of time and *V* is the volume of the system, we can say that the system is in a semiclassical TC phase. A more detailed explanation of the deduction of Eq. (5) and its relation to the criterion first proposed in^3^ can be found in the Supplemental Information, as well as the reasoning of validity of Eq. (5) being a criterion well-suited for the semiclassical mean-field study.

When dealing with one-dimensional systems, Eq. (5) can be reduced to

6 $$\begin{aligned} c(t) = \lim _{|\Delta x| \rightarrow \infty } \lim _{|t-t^\prime |\rightarrow \infty }\dfrac{1}{L_x}\int _{-L_x/2}^{L_x/2} \rho (x,t) \rho (x+\Delta x ,t^\prime ) dx, , \end{aligned}$$

where $L_x$ is the length of the system.

## Methods

In this section, we present the mathematical framework we will employ to investigate the dynamics of the condensate. First, we will present the equation that governs the evolution of the mean-field, which is a semi-classical and deterministic approach for the condensate. After that, we will present a way of going beyond the usual mean-field description by adding the effect of quantum uncertainty to the otherwise deterministic mean-field evolution. These quantum effects are, however, treated as corrections to the equation that governs the mean-field dynamics and are not sufficient to recreate the full range of quantum phenomena.

### Mean-field evolution

Here, we will present the modified Gross–Pitaevskii equation (GPE) which governs the mean-field dynamics BECs. The same equation is valid for the BEC of exciton–polaritons, and for the BEC of bare excitons, both under an external potential, $V_{\textrm{eff}} (r)$, caused by different reasons, as explained in the previous section. Both quasiparticles are described by a Hamiltonian $\hat{H}$ for a weakly interacting dilute Bose gas^37^:

7 $$\begin{aligned} \hat{H} = \int d^{2} r\ \ \hat{\psi }^{\dagger } (r) \left( - \frac{\hbar ^2\nabla ^{2}}{2M_{p}} + V_{\textrm{eff}} (r)\right) \hat{\psi } (r) + \frac{U_{\textrm{eff}}}{2} \hat{\psi }^{\dagger } (r) \hat{\psi }^{\dagger } (r) \hat{\psi } (r) \hat{\psi } (r), \end{aligned}$$

where $\hat{\psi }^{\dagger } (r)$ and $\hat{\psi } (r)$ are creation and annihilation Bose operators for the polaritons or excitons, $M_{p}$ is the effective mass of the quasiparticle, $V_{\textrm{eff}}(r)$ is the effective periodic potential acting on them. and $U_{\textrm{eff}}$ is the Fourier image of the pair polariton-polariton (or exciton–exciton) repulsion potential at zero momentum. For excitons, this repulsion is given by $U_{\textrm{eff}}=U_{\textrm{ex}}=\frac{3\hbar ^2}{M_{\textrm{ex}}}$, where $M_{ex}$ is the exciton mass; and $U_{\textrm{eff}}=U_{\textrm{pol}}=\frac{1}{4}U_{\textrm{ex}}$, for polaritons^37^.

The density matrix $\rho$ for non-equilibrium BEC can be obtained from the quantum Lindblad master equation^27^:

8 $$\begin{aligned} \frac{\partial \rho }{\partial t} = - \frac{i}{\hbar } \left[ \hat{H},\rho \right] + \int d^{2} r \left( \kappa \mathcal {L}\left[ \hat{\psi } (r), \rho \right] + \gamma \mathcal {L}\left[ \hat{\psi }^{\dagger } (r),\rho \right] + \frac{\Gamma }{2}\mathcal {L}\left[ \hat{\psi }^{2} (r),\rho \right] \right) , \end{aligned}$$

where the usual Lindblad superoperator is defined as

9 $$\begin{aligned} \mathcal {L}\left[ \hat{X},\rho \right] = 2 \hat{X}\rho \hat{X}^{\dagger } - \left[ \hat{X}^{\dagger } \hat{X}, \rho \right] _{+}, \end{aligned}$$

and $\kappa$, $\gamma$ and $\Gamma$ are the rates of single-particle loss, single-particle incoherent pumping and two-particle loss, respectively.

To properly obtain the time-evolution of the density matrix $\rho$ by solving Eq. (8) is an extremely complex mathematical problem. The usual approach is to, instead, study the semiclassical mean-field dynamics. The mean-field equation of motion can be obtained by replacing $\left\langle \hat{\psi } (r) \right\rangle = \tilde{\varphi } (r)$ (where $\tilde{\varphi } (r)$ is the wave function of the condensate) and decoupling all correlators. This procedure results in a modified Gross-Pitaevskii equation (GPE)^26^, including dissipative terms describing particle gain and loss:

10 $$\begin{aligned} i \hbar \frac{\partial \tilde{\varphi }}{\partial t} = \left[ - \frac{\hbar ^2\nabla ^{2}}{2M_{P}} + V_{\textrm{eff}} (r) + U_{\textrm{eff}}\left| \tilde{\varphi }\right| ^{2} + i \left( \gamma _{\textrm{eff}} - \Gamma \left| \tilde{\varphi }\right| ^{2}\right) \right] \tilde{\varphi }, \end{aligned}$$

where $\gamma _{\textrm{eff}} = \gamma - \kappa$ is the effective pumping rate.

When we consider our system to be a strip, with a width $L_y$ in the *y* direction much smaller than the size of $L_x$ of the strip in the *x* direction ($L_y\ll L_x$), we can treat Eq. (10) as one-dimensional, by making the substitution $\tilde{\varphi }(r)\rightarrow \dfrac{\varphi (x)}{\sqrt{L_y}}$, which leads to

11 $$\begin{aligned} i \hbar \frac{\partial \varphi }{\partial t} = \left[ - \frac{\hbar ^2}{2M_{P}}\dfrac{\partial ^2}{\partial x^2} + V_{\textrm{eff}} (x) +\dfrac{ U_{\textrm{eff}}}{L_y}\left| \varphi \right| ^{2} + i \left( \gamma _{\textrm{eff}} - \dfrac{\Gamma }{L_y} \left| \varphi \right| ^{2}\right) \right] \varphi . \end{aligned}$$

In our one-dimentional approximation for narrow strips, we have $V_{\textrm{eff}} (x)$ sinusoidal for both systems, namely, polariton BEC in a TMDC strip, embedded in a microcavity, and exciton BEC in a strip of a twisted TMDC bilayer, for very different reasons. In the polaritonic system, this potential is created by the special manufacturing of the microcavity, while for the excitonic system, the potential arises naturally from the relative twist of the layers, as explained in the previous section.

### Beyond mean-field description

Here, we explain what corrections should be added to Eq. (11), in order for us to consider some of the effects of quantu randomness to the otherwise deterministic mean-field evolution.

12 $$\begin{aligned} i \hbar \frac{\partial \varphi _{C}}{\partial t} = \left[ - \frac{\hbar ^{2} \nabla ^{2}}{2M_P} + V_{\textrm{eff}} (\textbf{r}) + U_{\textrm{eff}}\left| \varphi _{C}\right| ^{2} + i \left( \gamma _{\textrm{eff}}-\Gamma |\varphi _{C}|^2 \right) \right] \varphi _{C} + i \left( \kappa + \gamma \right) \varphi _{Q}, \end{aligned}$$

where $\varphi _{Q}$ is the “quantum” field, which can be represented by the dissipative-stochastic GPE (DSGPE). The DSGPE is equivalent to Eq. (12) with the replacement $i \left( \kappa + \gamma \right) \varphi _{Q} \rightarrow \xi (\textbf{r},t)$, where $\xi (\textbf{r},t)$ represents a Gaussian white noise process with

13 $$\begin{aligned} \left\langle \xi (\textbf{r},t) \right\rangle = 0, \hspace{1cm} \left\langle \xi (\textbf{r},t) \bar{\xi } (\textbf{r}^{\prime },t^{\prime }) \right\rangle = \frac{\left( \gamma + \kappa \right) }{2}\delta \left( t - t^{\prime }\right) \delta \left( \textbf{r} - \textbf{r}^{\prime }\right) . \end{aligned}$$

For the one-dimensional case we are considering, the DSGPE becomes

14 $$\begin{aligned} i \hbar \frac{\partial \varphi }{\partial t} = \left[ - \frac{\hbar ^2}{2M_{P}}\dfrac{\partial ^2}{\partial x^2} + V_{\textrm{eff}} (x) +\dfrac{ U_{\textrm{eff}}}{L_y}\left| \varphi \right| ^{2} + i \left( \gamma _{\textrm{eff}} - \dfrac{\Gamma }{L_y} \left| \varphi \right| ^{2}\right) \right] \varphi + \xi (x,t), \end{aligned}$$

where the sub-index *C* of $\varphi _C$ was omitted for simplicity.

One thing that is important to note, however, is that in reaching the DSGPE represented in Eq. (14), quantum phenomena are treated perturbatively. The effects of the addition of the quantum noise term, $\xi (x,t)$, do not fully capture all quantum aspects pertinent to the system. Such a term is only efficient in mimicking local variations to order parameters due to inherent quantum randomness, but fail to show, for example, effects caused by quantum correlations between two different measures. In order to properly evaluate all quantum effects, one would have to obtain the full dynamics of the system’s density matrix, obtained from Eq. (8), which is a very complicated mathematical problem, as previously discussed.

## Numerical results

In this section, we will present our numerical results. These results will be separated as such: first, we will do an in-depth analysis of the time evolution of the BEC of exciton–polaritons inside a spatially curved optical microcavity, where we will prove that such a system is in a TC phase both when we consider just the mean-field evolution, and when we go beyond the usual mean-field description. After that, we will analyze the evolution of the BEC of bare excitons on a twisted TMDC bilayer and show that the same results still apply.

### Polariton BEC on a spatially curved cavity

First, we will consider a system of exciton–polaritons inside a spatially curved microcavity, as depicted in Fig. 1. We consider the length of the cavity $L_C(x)$ to be given by Eq. (2). We considered a strip of MoSe$_2$, in which the polaritons have an effective mass $M_P = 5.8 \times 10^{-6} m_0$, where $m_0$ is the free electron rest mass. The strip is inside a microcavity assembled with constituent elements similar to ones such as that in Ref.^48^, which has a refractive index $n=2.2$ and a mean width $L_0 = 2.3$ $\upmu$m. We consider the $q=5$ mode of that cavity, which resonates with the excitons in the MoSe$_2$ strip. The effective Rabi coupling between photons and excitons is $\hbar \Omega = 20.0$ meV. We have chosen the period of the effective potential, $a_{C}$, to be $a_C=$10 $\upmu$m. Throughout all of our simulations, we considered the length of the strip to be $L_x = 4000$ $\upmu$m, and its width to be $W= 1$ $\upmu$m. We consider the effective pump rate to be $\gamma _{\textrm{eff}} = 0.1$ meV, and the two-particle loss is assumed to be $\Gamma \approx 0.3$ $U_{\textrm{eff}}$^49^.

Our results showed that the polariton BEC density , $P(x,t) = |\varphi (x,t)|^2$, oscillates around the value, $P_0 = \dfrac{\gamma _{\textrm{eff}} L_y}{\Gamma }\approx 69.8 \times 10^3$ $\upmu$m$^{-1}$, which is the condensate density for the system being in the steady state in the case of an absence of external potential. For almost all the plots the system was chosen to begin the simulation at $t=0$ in the steady-state of the unperturbed system, $\varphi (t=0,x)= \sqrt{P_0}$, the only exception being the ones in Fig. 5b. We will discuss this in further detail as we analyze each of the plots.

Our results are divided into three subsections. The first one deals with the mean-field dynamics, represented by the GPE in Eq. (11). In the second one we consider the mean-field dynamics obeying to a modified version of the GPE given by Eq. (11), assuming the polariton decay rate $\kappa$ to be spatially dependent, and not constant. The last subsection provides the results of the polariton condensate dynamics beyond the mean-field description, when the polariton BEC time-evolution is governed by the DSGPE presented by Eq. (14). Another set of results is shown in the Supplemental Information, in which we consider both the time evolution of the BEC phase, as well as that of the polariton reservoir. In such considerations, the polariton BEC will evolve following another modified version of the GPE in Eq. (11), in which interactions between the BEC and non-BEC polaritons are taken into consideration, as well as transitions between both polaritonic phases being allowed. In the Supplemental Information, we consider both a constant pump of reservoir polaritons as well as a spatially varying pump with the same period as the external potential on the polaritons. We show that the results there are completely akin to those of a constant effective pumping rate, as well as a spatially varying pumping rate, that will be shown here.

#### Mean-field evolution

**Figure 3:**
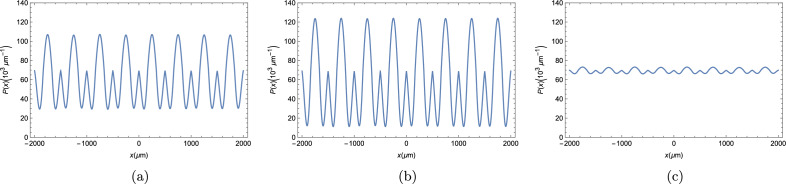
Polariton BEC density $P(x,t) = |\varphi (x,t)|^2$ for the polariton BEC in a ribbon of length 4000 $\upmu$m at three arbitrary times times, each plot is taken 5 ps after the previous. (**a**) t = 3005 ps ; (**b**) t = 3010 ps; and (**c**) t = 3015 ps.

Our first result depicts the polariton BEC density throughout the strip at three different times. We allowed the simulation to run a long time before taking those plots in order to be sure that, if our system was able to thermalize and reach a steady state, it would have done so. By looking at Fig. 3, we see that the polariton BEC density throughout the strip is oscillating in time. We can see that this condensate appears to be pulsating around the steady state density for a planar microcavity, $P_0\approx 69.8 \times 10^3$ $\upmu$m$^{-1}$, since it deviates from this constant density a moderate amount at Fig. 3a, then it approaches $P_0$ throughout the entire strip af Fig. 3b and deviates even more than on Fig. 3a on Fig. 3c. Since the time difference between each of the plots in Fig. 3 is of 5 ps, the period of these oscillations cannot be longer than just a few ps, meaning that our chosen start time of 3 ns is, indeed, sufficiently big to discard the possibility of the system thermalizing. The inability of reaching a steady state is one condition for the system to be a TC, which serves as a first evidence of this phase^2^.

**Figure 4:**
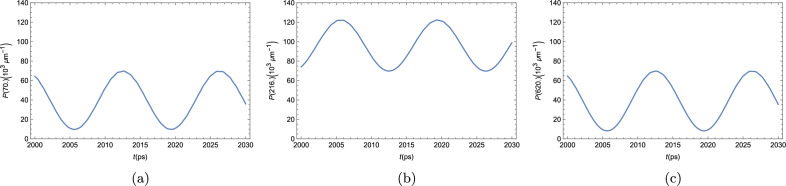
Polariton BEC density $P(x,t) = |\varphi (x,t)|^2$ as a function of time at three arbitrary positions within the strip.

In Fig. 4, we see the time evolution of the polariton BEC density in three different positions in the strip. The condensate density oscillates between $P_0$ and a value that can be smaller than $P_0$, as in Fig. 4a,c, or greater than $P_0$, as in Fig 4b. The system oscillates in phase, reaching $P_0$ at the same time for all positions; and reaching the maximum deviation also at the same time. The period of oscillation can be seen to be of around 3 ps. We, again, see that the system does not show any tendency of reaching a steady state.

**Figure 5:**
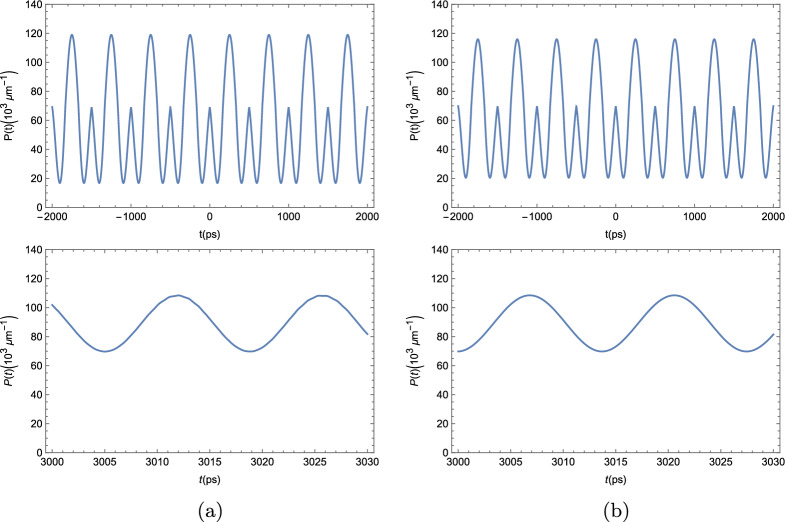
Comparison between the dynamics of the BEC of exciton–polaritons for two different initial conditions. On the top, we see the condensate distribution throughout the entire strip at an arbitrary time, long after the beginning of the simulation; on the bottom, we see the time evolution for the condensate density at $x= 125$ $\upmu$m for a period of 5 ps for the system starting at (**a**) the steady-state solution for the unperturbed condensate, $\varphi (x,t=0) = \sqrt{\dfrac{\gamma _{\textrm{eff}} L_y}{\Gamma }}$; and (**b**) the vacuum state, $\varphi (x,t=0)=\epsilon$, with $\epsilon$ infinitesimal.

In order to check if our result depends on the initial conditions chosen by us, namely the state $\varphi _0(x) = \sqrt{\dfrac{(\gamma -\kappa )L_y}{\Gamma }}$, which is the steady-state for the unperturbed BEC (when $V_{\textrm{eff}}=0$), we tried a different initial condition, when the system started in the vacuum state. This result is shown in Fig. 5.

On the top row of Fig. 5, we see a picture of the condensate density throughout the entire strip at an arbitrary time, just like one of the panels in Fig. 3. On the bottom row, we see the time evolution of the condensate density at an arbitrary position, just like in of the panels in Fig. 4. In Fig. 5a, the system started at $t=0$ in the steady-state of the unperturbed system, like on all previous and future Figures, while in Fig. 5b, the system started in the vacuum state. It is evident that the only difference between the figures is the overall phase of the system. The dynamics themselves, for *t* long enough, are completely equivalent. This means that our results were not impacted by our choice of initial state and should be verifiable regardless of how the system is at $t=0$.

**Figure 6:**
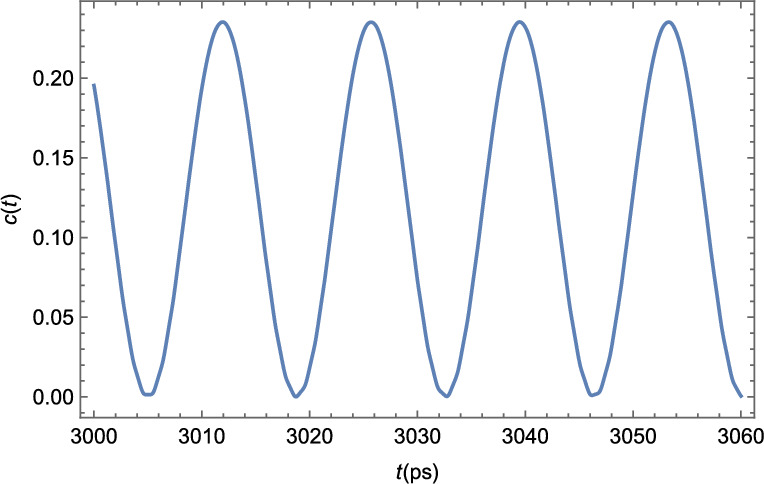
Numerical results for the two-point correlation function *c*(*t*) as defined in Eq. (6) for the polariton BEC. For this plot, we considered the order parameter $\rho$ to be the relative deviation between the condensate density *P*(*x*, *t*) and the unperturbed steady-state density $P_0$, namely, $\rho (x,t) = \dfrac{P (x,t)-P_0}{P_0}$. We took $x-x^\prime$ to be half the length of the strip. The limit $|t-t^\prime |\rightarrow \infty$ was taken by fixing $t^\prime$ at $t^\prime = 100$ ps, and assuming *t* to vary between 3000 and 3060 ps. This way $|t-t^\prime | \gg \tau _C$, where $\tau _C \approx 14$ ps is the period of the condensate oscillations.

So far, we have shown evidence that our system *could* be in a TC phase, but haven’t yet provided the definite proof, through the study of the mathematical criterion for semiclassical TCs proposed in Eq. (6). This will be shown in Fig. 6.

We consider the order parameter $\rho$ to be the relative deviation of the BEC density, *P*(*x*, *t*), from the unperturbed steady-state density $P_0 = |\varphi _0|^2$, $\rho (x,t) = \dfrac{P (x,t)-P_0}{P_0}$. Since *P*(*x*, *t*) is a valid order parameter for BECs, $\rho (x,t)$ is also one, given that $P_0$ is a constant value.

It can be seen from Fig. 6 that the polariton BEC in a strip of TMDC, embedded in a microcavity in the presence of the external periodic potential, in fact, obeys the mathematical condition given by Eq. (6) within the mean-field approach, and can be, therefore, characterized as a Time Crystal. A brief comparison between the two-point correlator shown in Fig 6 with the time evolution of the polariton condensate shown in Fig. 4 shows us that this correlator oscillates in time with the same frequency as the condensate itself oscillates, a result that does seem reasonable.

#### Spatially-varying loss

**Figure 7:**
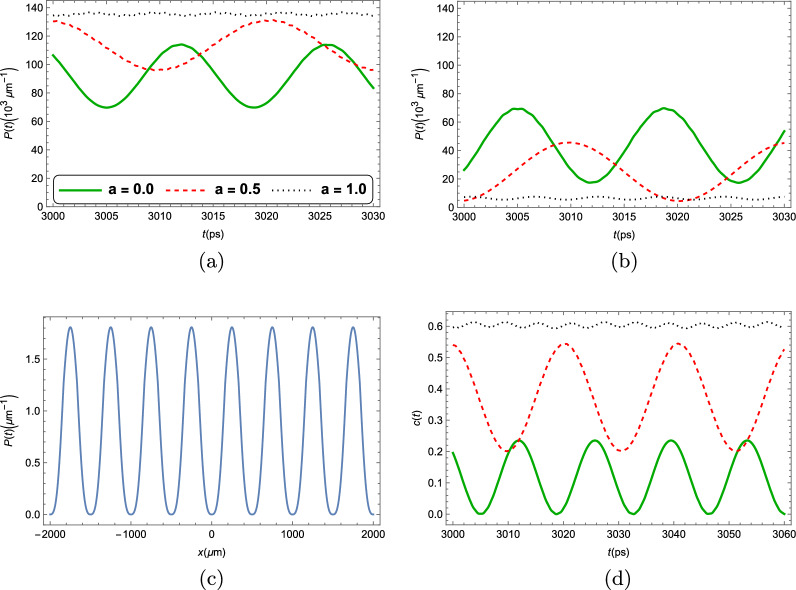
(**a,b**) Time evolution of the polariton mean-field condensate density at two arbitrary positions in the strip, with a spatially varying effective gain term $\gamma _{\textrm{eff}} \rightarrow \gamma _{\textrm{eff}}\left( 1-a\cos (kx)\right)$. The solid green line has $a=0$, the dashed red line has $a = 0.5$, and the dotted black line has $a= 1.0$. (**c**) Steady-state density of the polariton condensate mean-field for the $a=1$ case. (**d**) The two-point correlator given by Eq. (6).

So far, all of our results were given directly by the numerical solution of Eq. (11), in which the gain and loss of particles, given by $\kappa$ and $\gamma$, are considered to be constant throughout the strip. One might wonder, however, whether this is the best approximation for a system that is subject to an external periodic potential. Given that the average lifetime of photons in a microcavity is usually usually a decreasing function of their energy, it is only reasonable to assume that the loss ratio should be higher, whenever the energy of photons is higher. In this section, we study the effects of a spatially varying loss in the time-evolution of the polariton condensate, and of the two-point correlator *c*(*t*), given by Eq. (6). In the following results, we replace the polariton decay rate, $\kappa$ by $\kappa \left( 1 + a^\prime \cos (kx)\right)$, where $k = 2\pi /a_{p}$ with $a_{p}$ being the same period as that of the external potential $V_{\textrm{eff}}(x)$, and $a^\prime < 1$ is a numeric parameter that modulates the intensity of this spatial dependence. This leads to a spatially dependent effective gain ratio $\gamma _{\textrm{eff}}\rightarrow \gamma _{\textrm{eff}}\left( 1-a\cos (kx)\right)$, in which $a = \dfrac{\gamma }{\kappa +\gamma }a^\prime$. In Fig. 7, we study how the time-evolution of the condensate mean-field density and the two-point correlator *c*(*t*) varies with the parameter *a*.

By analyzing Fig. 7, it is evident that the substitution $\gamma _{\textrm{eff}} \rightarrow \gamma _{\textrm{eff}}\left( 1 - a\cos (kx)\right)$ in the GPE of Eq. (11) significantly impacts the dynamics. When compared to the constant gain/loss case (when $a = 0$), being subject to a spatially dependent gain/loss changes the period of oscillation of the overall condensate. This can be seen by the comparison of the solid green lines and dotted red lines from the first two panels of Fig. 7. Furthermore, it changes the amplitudes of the oscillations and the average value of the condensate density at each given point. When the value of *a* reaches $a=1$, we stop seeing time-oscillations in the condensate density and the system reaches a final steady state, which is depicted in Fig. 7c.

Overall, we see that the effect of a spatially dependent loss, in which gain is maximal (minimal) where energy of the particles is minimal (maximal), is to diminish the time oscillations in the condensate density that serves as evidence of a TC phase. However, a semiclassical TC phase is still observed, given that this spatial dependence is somewhat weak. This can be seen on Fig. 7d, where the two-point correlator *c*(*t*) from Eq. (6) is depicted. It is clear that, in the $a = 0.5$ case, the overall oscillations of *c*(*t*) are still evident, being even more intense than those of the $a = 0$ case. The same cannot be said of the $a = 1$ case, in which *c*(*t*) appears to be almost constant, destroying any evidence of time crystallization.

#### Beyond mean-field description

**Figure 8:**
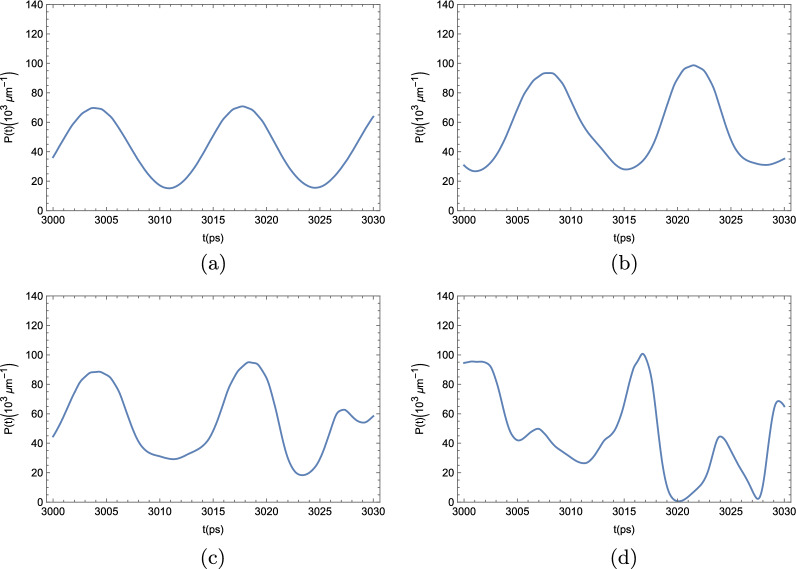
Polariton BEC density $P(x,x) = |\varphi (x,t)|^2$ at an arbitrary position in a strip 4000 $\upmu$m long for different values of $\kappa$ and $\gamma$, when quantum uncertainty is taken into consideration. In all the simulations, the effective pumping rate $\gamma _{\textrm{eff}} = \gamma - \kappa$ was kept constant and considered to be $\gamma _{\textrm{eff}} = 0.1$ meV. (**a**) $\kappa + \gamma = 1$ meV; (**b**) $\kappa + \gamma =$ 5 meV; (**c**) $\kappa + \gamma =$ 10 meV; and (**d**) $\kappa + \gamma =$ 30 meV.

We now turn our attention to the BEC dynamics when quantum corrections are taken into consideration. The results we show here depict the numerical solution of the time evolution of the polariton BEC density following the DSGPE in Eq. (14). In Fig.  8, we see the time evolution of the condensate density in various positions along the ribbon, similarly to Fig. 4, for the mean-field approach.

**Figure 9:**
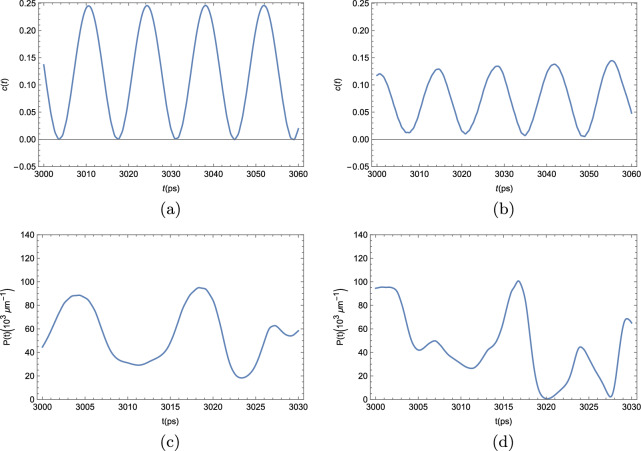
Two-point correlation function *c*(*t*) as defined by Eq. (6) for the exciton-polariton condensate, with the addition of quantum noise. Like in Fig. 6, we considered the order parameter $\rho$ to be the relative deviation between the condensate density *P*(*x*, *t*) and the unperturbed steady-state density $P_0$, namely, $\rho (x,t) = \dfrac{P (x,t)-P_0}{P_0}$. We took $x-x^\prime$ to be half the length of the strip. The limit $|t-t^\prime |\rightarrow \infty$ was taken by fixing $t^\prime$ at $t^\prime = 100$ ps, and taking *t* to vary between 1000 and 1020 ps. This way $|t-t^\prime | \gg \tau _C$, where $\tau _C \approx 14$ ps is the period of the condensate oscillations. In all the simulations, the effective pumping rate $\gamma _{\textrm{eff}} = \gamma - \kappa$ was kept constant and considered to be $\gamma _{\textrm{eff}} = 0.1$ meV. (**a**) $\kappa + \gamma = 1$ meV; (**b**) $\kappa + \gamma =$ 5 meV; (**c**) $\kappa + \gamma =$ 10 meV; and (**d**) $\kappa + \gamma =$ 30 meV.

As it can be seen from the panels of Fig. 8, the addition of the noise term in the DSGPE significantly interferes with the dynamics. If the noise term is sufficiently small, clear and well defined oscillations can still be seen, as in panel (a). As the noise term grows, meaning greater pump and decay rates $\gamma$ and $\kappa$, which were increased while maintaining $\gamma _{\textrm{eff}} = \gamma - \kappa$ constant at $\gamma _{\textrm{eff}} = 0.1$ meV. In panel (b), $\gamma$ and $\kappa$ are suffiently big to visibly affect the otherwise perfect oscillations in the condensate density, but the overall shape is still maintained. In panel (c), this shape is further deformed; and, in panel (d), the incoherent pumping and decay rates are big enough as to make the oscillations on the condensate density appear to be completely random. In the next plots, depicted in Fig. 9, we turn our attention to the effects of the stochastic corrections to the GPE in the two-point correlator defined in Eq. (6).

When we analyze the time evolution of the two-point correlator *c*(*t*) beyond the mean-field description, we reach a similar conclusion as to when we study the time-evolution of the condensate density profile. Provided that the decay and pump rates are sufficiently small, we barely notice any deviation from the mean-field case, as can be seen in Fig. 9a. However, as $\kappa +\gamma$ grows, the otherwise well-defined shape of *c*(*t*) starts to deform (panels (b), and (c)), until it becomes completely unrecognizable, and, effectively, completely random (panel (d)).

**Figure 10:**
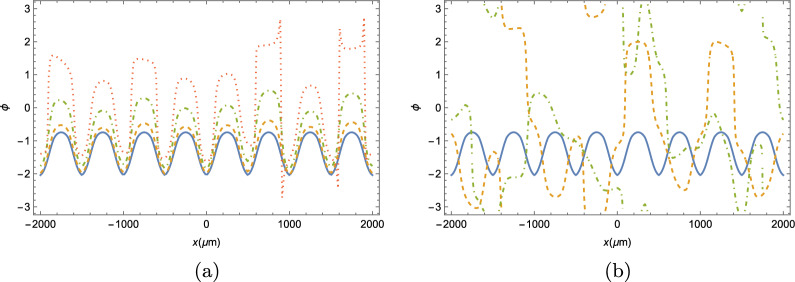
Phase of the condensate wavefunction throughout the strip at an arbitrary long time $t\gg \tau _C$, where $\tau _C \approx 14$ ps is the period of oscillations of the BEC. (**a**) Solid blue line corresponds to the mean-field dynamics, dashed yellow line corresponds to $\kappa + \gamma = 0.5$ meV, the dot-dashed green line corresponds to $\kappa + \gamma = 1$ meV, and the dotted red line corresponds to $\kappa + \gamma = 1.5$ meV. (**b**) the solid blue line corresponds to the mean-field dynamics, the solid blue line corresponds to the mean-field dynamics, the dashed yellow line corresponds to $\kappa + \gamma = 2$ meV, and the dot-dashed green line corresponds to $\kappa + \gamma = 5$ meV.

In addition, we present the time evolution of the phase of the condensate wave function. It is expected that the addition of a noise term should disturb the distribution pattern of the phase throughout the strip. In the plots of Fig. 10, we show how the phase profile of the condensate evolves for different values of the quantum noise $\kappa +\gamma$.

As it can be seen from the panels of Fig. 10, provided that the quantum noise is sufficiently small, the phase throughout the strip behaves in a similar manner than in the mean-field approximation, as can be seen in panel (a). The actual value of the phase does show some random variance from the mean-field phase. The variance is more intense the bigger the noise is, however, the overall shape is maintained with local maxima and minima of the phase occuring in the same places. If the noise increases, however, the overall phase of the wave-function will, eventually, become a seemingly random function of the position, as can be seen in panel (b). It is interesting to note, however, that this seemingly random phase occurs even when the two-point correlator *c*(*t*) still shows a moderately well-defined shape.

### Excitonic BEC on a twisted TMDC bilayer

**Figure 11:**
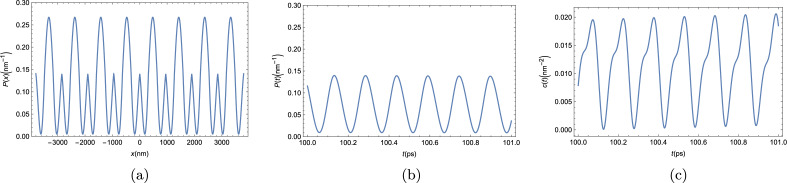
Numerical results for the dynamics of the excitonic condensatein a twisted TMDC bilayer in the mean-field approach. (**a**) Time evolution of the condensate density at position $x= 100$ nm; (**b**) condensate density throughout the entire strip at $t=3$ ns and (**c**) two-point correlator as defined in Eq. (6).

**Figure 12:**
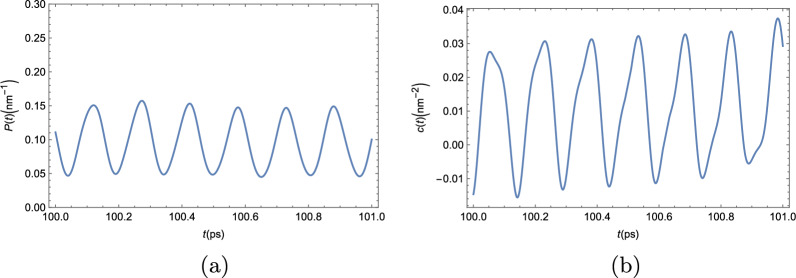
Numerical results for the dynamics of the excitonic condensate in a twisted TMDC bilayer beyond the mean-field approach. (**a**) Condensate density in an arbitrary position of the strip. (**b**) Two-point correlator defined by Eq. (6).

We will now present our results for the mean-field dynamics of the bare exciton BEC in a strip of twisted TMDC bilayer. In order to avoid seemingly repetitive plots and discussions, we will compile all results for this condensate in two figures. In Fig. 11, we combine all the results for the mean-field dynamics of the condensate. In Fig. 12, we present our results for the dynamics of the condensate when quantum uncertainty is taken into consideration.

We considered the same system as in Ref.^40^, namely a WSe$_2$/MoSe$_2$ bilayer heterostructure twisted by 1$^\circ$, we will have $M_{ex}\approx 0.84$ $m_0$, $a_M\approx 19$ nm and $V_0\approx 18$ meV. We considered our strip to be 8 $\mu$m long and to have a width of 10.4 nm, which obeys the criterion for the effective potential to be given by Eq. (4). As with the polaritonic system, we considered $\gamma _{\textrm{eff}} = 0.1$ meV, and $\Gamma \approx 0.3$ $U_{\textrm{eff}}$^49^. The resulting dynamics of the excitonic condensate and two-point correlator can all be seen in Fig. 11.

Each of the panels in Fig. 11 shows results for the mean-field evolution of the excitonic BEC in a twisted TMDC bilayer that are similar to one of the figures in the mean-field analysis of the polariton BEC. Figure 11a shows the BEC mean-field density throughout the entire strip, just like Fig. 3 did for the polaritons. The similarities are evident, the overall shape of the BEC is completely equivalent, the only difference being the scale. This comes from the fact that the bare exciton mass is about six orders of magnitude larger than the effective mass of the polaritons, which leads to the bare exciton BEC length scales to be about three orders of magnitude smaller than those of the polariton BEC. Figure 11b shows the time evolution of the condensate mean-field density in an arbitrary position as a function of time, just like what we saw in the panels of Fig. 4 for the polariton BEC. Those results are completely equivalent, just with a different period of oscillation, which is about one order of magnitude smaller. Lastly, in Fig. 11c, we show the two-point correlator from Eq. (6). It is evident that this correlator also obeys the semiclassical TC criterion, given in Eq. (6), and the exciton BEC in a twisted TMDC bilayer is, therefore, a TC.

In Fig. 12, we see numerical results for the time-evolution of the condensate density in an arbitrary position (panel (a)); and the two-point correlator defined in Eq. (6) (panel (b)). Just like in the polaritonic condensate beyond the mean-field description, we lose the perfect definition of the oscillations when quantum uncertainty is taken into consideration. The actual height of each peak and valley in Fig. 12a is slightly different from one another. This, however, is not nearly enough to erase the overall shape seen by the mean-field dynamics in Fig. 11b. The comparison between the two-point correlator beyond mean-field shown in Fig. 12b with its mean-field counterpart, shown in Fig. 11c is also very similar to the polaritonic condensate case. The overall shape of this correlator does change visibly, but its shape still shows clear signs of oscillations in time. The intensity of the peaks was greatly enhanced in the non-deterministic beyond mean-field approach, when compared to the deterministic mean-field approach and, like in the polaritonic BEC, we now see negative values for the correlator that were not observed in the mean-field approach. However, the criterion proposed in Eq. (6) is still obeyed and the system is, therefore, still in a TC phase. As we can see from the panels of Fig. 12, the results obtained for the mean-field dynamics of the excitonic BEC still hold even when quantum uncertainty is taken into consideration, just like for the polaritonic BEC.

## Concluding remarks

Throughout this paper we have studied two distinct BEC systems, namely, excitons in a twisted TMDC bilayer and exciton–polaritons in a strip of MoSe$_2$ embedded in a microcavity with a spatial curvature, causing the effective periodic potential. Our results show that both systems are good candidates for time crystallization. We have shown that the mean-field density profiles of both systems are periodic functions of time, and that both systems satisfy the semiclassical TC criterion, by exhibiting long-range non-trivial time-dependent correlations of the order parameter. Our results were robust enough to survive, even when quantum corrections were added to the mean-field description provided that the polaritonic or excitonic gain and loss ratios are sufficiently small. We have shown, however, that a spatially varying loss relation with the same period as the external potential diminishes the density oscillations and can even completely destroy them, provided that this spatial dependence is strong enough.

It is our opinion that both of our considered systems are good candidates for future experimental verification. Since recently a BEC of exciton-polaritons has been verified in room-temperature settings in halide perovskite^28^, it is reasonable to assume the same might be possible for TMDCs. If that is the case, our results lead to the possibility of having a room-temperature TC, which would depend only on the manufacturing of our proposed spatially curved microcavity. Our second system, namely bare-excitons on a twisted TMDC bilayer, on the other hand, has been seen to form condensates at temperatures around 190 K^23^, way above the ultra-cold regime in which atoms form condensates and reachable in most research settings. Such a system has the advantage that the external potential arises naturally from the twisting of the TMDC layers, forming the bilayer^40^, making the manufacture process much simpler.

### Supplementary Information

Supplementary Information.

## Data Availability

All data generated or analyzed during this study are included in this published article.
