# Supplementary material for: Polaritonic and excitonic semiclassical time crystals based on TMDC strips in an external periodic potential
Source: Sci Rep. 2023 Nov 11;13:19707. doi: 10.1038/s41598-023-46077-0 (PMC10640621; doi:10.1038/s41598-023-46077-0)
Supplement: Supplementary file 1 — Supplementary Information. [file 41598_2023_46077_MOESM1_ESM.pdf]

Suplemental Information for Polaritonic and Excitonic Semiclassical Time Crystals based on TMDC strips in an external periodic potential

I. MATHEMATICAL CRITERION FOR TIME CRYSTALS

A. Criterion for a Quantum Time Crystal

Here, we present the proper mathematical criterion for a quantum time crystal. A time crystal (TC) is a phase of matter that has been proposed in 2012 by Wilczek [1] and has been the object of much interest since then.

Like many other phases of matter, TCs arise when the system spontaneously breaks one of the symmetries of its Hamiltonian. When a system spontaneously breaks, for example, translational symmetry, it becomes a crystal; if it breaks spin rotational symmetry, it becomes a magnet. TCs, on the other hand, appear when a system breaks the time translational symmetry (TTS).

In 2015, Watanabe and Oshikawa provided a mathematical criterion to verify whether a system is in a quantum time crystal phase [2]. In it, the authors followed steps based on the mathematical definition of a spatial crystal. They say that, for the system to be considered a crystal, it has to show a non-trivial two-point correlator in an order parameter $\hat{O}$ in space far apart $\left(\lim_{|\mathbf{r}-\mathbf{r}'|\to\infty}\langle\hat{O}(\mathbf{r})\hat{O}(\mathbf{r}')\rangle = f(\mathbf{r}-\mathbf{r}')\right)$. Analogously, for a system to be a TC, it has to show some non-trivial long-range correlations in time for some order parameter $\hat{O}$ at two different and long-apart times, namely,

$$\lim_{|\mathbf{r}-\mathbf{r}'|\to\infty} \lim_{t-t'\to\infty} \left\langle \hat{O}(\mathbf{r},t)\hat{O}(\mathbf{r}',t') \right\rangle = c(t), \tag{1}$$

for some non-trivial, or, in other words, non-stationary, function $c(t)$.

It is important to note that, in order for the correlator proposed in Eq. (1) to be evaluated, one needs to know the proper time evolution of the density matrix of the studied system. This is not always an easy task. In this paper, we instead studied the mean-field evolution of the condensates. In the mean-field approach, one cannot analyze the criterion exactly as proposed in Ref. [2]. As a consequence of that, we will present the semiclassical modification of Eq. (1), which will serve as the criterion for semiclassical time crystals. The modified criterion, unlike the one in Eq. (1), can be used to identify TTS breaking in the mean-field approach.

B. Criterion for Semiclassical Time Crystals

Here, we present the criterion which is suited for the study of non-trivial time correlations in the mean-field dynamics and can be used to verify whether a system behaves as a semiclassical time crystal or not.

In Ref. [2], Watanabe and Oshikawa were focusing their studies entirely on strictly quantum time crystals. For that reason, we see in Eq. (1) the expected value of a product of order parameters which are quantum observables at different times and positions, represented by $\hat{O}$. In order to calculate the expected value in Eq. (1) one would, therefore, need to know the full density matrix of the system at both times, which might not always be an easy task. By replacing the system's density matrix by the mean-field wave function, one ends up losing all information on strictly quantum correlations. Therefore, it is impossible for us to compute precise expected values of a product of two quantum observables, as proposed by Watanabe and Oshikawa. However, one can still extract the time evolution of some order parameters from the mean-field dynamics. This is the case, for example, of the condensate mean-field density, which is the order parameter usually considered in the study of BECs.

Here, we modify the correlator proposed by Watanabe and Oshikawa by replacing the strictly quantum order parameter $\hat{O}(\mathbf{r},t)$ by a semiclassical order parameter $\rho(\mathbf{r},t)$, which must depend on the mean-field wave function $\varphi(\mathbf{r},t)$. Namely, we replace $\left\langle \hat{O}(\mathbf{r},t)\hat{O}(\mathbf{r}',t') \right\rangle \to \langle\rho(\mathbf{r},t)\rho(\mathbf{r}',t')\rangle$ in Eq. (1). In this case, the quantum expectation value is replaced by the average over all space, while keeping $\mathbf{r}-\mathbf{r}' \equiv \Delta\mathbf{r}$ constant. In other words, the semiclassical analogue to the criterion shown in Eq. (1) is

$$\lim_{|\mathbf{r}-\mathbf{r}'|\to\infty} \lim_{|t-t'|\to\infty} \langle\rho(\mathbf{r},t)\rho(\mathbf{r}',t')\rangle = \lim_{|\Delta\mathbf{r}|\to\infty} \lim_{|t-t'|\to\infty} \frac{1}{V}\int \rho(\mathbf{r},t)\rho(\mathbf{r}+\Delta\mathbf{r},t')d\mathbf{r} = c(t), \tag{2}$$

where V is the system's volume, and $t' \ll t$ is an arbitrary time. The order parameter ρ could be, for example, the average local magnetization for a magnetic system, or the mean-field condensate density. A system that obeys the criterion in Eq. (2) shows time-dependent two-point correlations that spontaneously break TTS and is, therefore, a time crystal, albeit not necessarily a quantum time crystal since correlations caused by strictly quantum phenomena will be completely missed by this analysis. Following the nomenclature of Refs. [3, 4], both of which have conducted studies of TCs within the mean-field approximation, we call TCs that obey the criterion in Eq. (2) to be Semiclassical Time Crystals.

II. NUMERICAL RESULTS FOR A POLARITON BEC COUPLED TO A POLARITON RESERVOIR

In the main-body of the paper, we have considered a polariton BEC whose mean-field dynamics obeys a Gross-Pitaevskii equation given by (Eq.(11) in the main text)

$$i\hbar\frac{\partial\varphi}{\partial t} = \left[-\frac{\hbar^2}{2M_P}\frac{\partial^2}{\partial x^2} + V_{\text{eff}}(x) + \frac{U_{\text{eff}}}{L_y}\left|\varphi\right|^2 + i\left(\gamma_{\text{eff}} - \frac{\Gamma}{L_y}\left|\varphi\right|^2\right)\right]\varphi. \tag{3}$$

Such an assumption assumes that polaritons are being created directly in the BEC phase, and is somewhat simplistic. A more accurate description would be to consider both the polariton BEC and a polariton reservoir, which, just like the BEC, can vary it's density on time and space [5]. In that case, the effective pump rate γ_{eff} is replaced by a term that couples the BEC to the reservoir, and is responsible for the creation of polaritons in the BEC phase. The time evolution of the polariton mean-field wave function will be governed by

$$i\hbar\frac{\partial\varphi}{\partial t} = \left[-\frac{\hbar^2}{2M_P}\frac{\partial^2}{\partial x^2} + V_{\text{eff}}(x) + \frac{U_{\text{eff}}}{L_y}\left|\varphi\right|^2 + i\left(Rn_R(x,t) - \kappa - \frac{\Gamma}{L_y}\left|\varphi\right|^2\right)\right]\varphi, \tag{4}$$

where $n_R(x,t)$ is the reservoir density, κ is the decay rate for BEC polaritons, and R is responsible for the relaxation of reservoir polaritons into the BEC phase. The time evolution of the reservoir density is, in turn, governed by

$$\frac{\partial n_R}{\partial t} = \mathcal{P} - \frac{1}{\hbar}\left(\gamma_R + R\left|\varphi\right|^2\right)n_R, \tag{5}$$

where γ_R is the decay rate for polaritons in the reservoir, and $\mathcal{P}$ is the pumping rate of polaritons into the reservoir.

A. Numerical Results

We will now show how the density profile of both the condensate phase, $P(x,t) = |\varphi(x,t)|^2$, and of the reservoir $n_R(x,t)$, evolve on time and space. We will consider both the case in which the pump rate $\mathcal{P}$ is constant throughout space, and when it is a periodic function in space, with the same period as that of the external potential $V_{\text{eff}}(x)$. We will also analyze the two-point correlator in both cases, and show that the system still obeys the semiclassical TC criterion, even when the finite polariton reservoir is taken into consideration. In our calculations, we considered $\kappa = 0.5$ meV, $\gamma_R = 2$ meV, $R = 0.05$ meV μm, and $\mathcal{P} = 63,660$ μm^{-1} ps^{-1} [6].

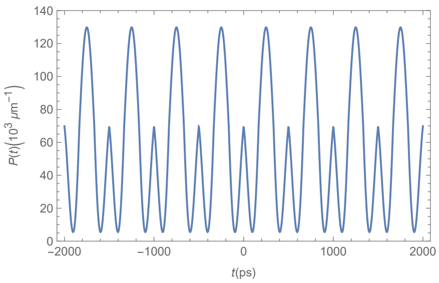

(a)

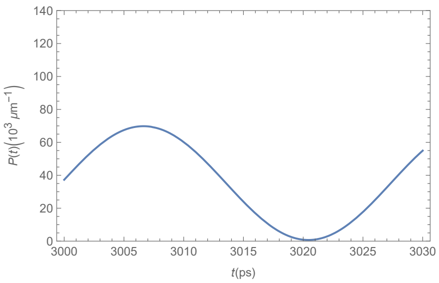

(b)

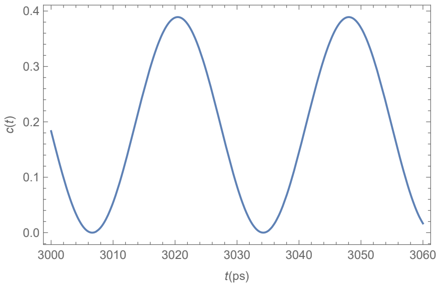

(c)

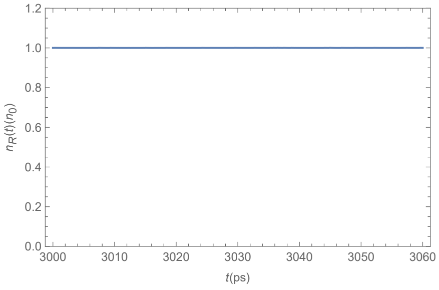

(d)

Figure 1: (a) Polariton BEC density profile throughout the strip at an arbitrarily long time. (b) Time evolution of the polariton BEC density at an arbitrary position in the strip. (c) Two point correlator $c(t)$, as defined in Eq. (2), for the order parameter $\rho(x,t) = \dfrac{P(x,t) - P_0}{P_0}$. (d) Time evolution of the density of the polariton reservoir n_R at an arbitrary position in units of the steady-state solution for the case without an external potential, n_0.

By analyzing the plots in Fig. 1, we see that in the mean-field description of our considered system, taking the interaction of the BEC of polaritons with the polariton reservoir, appears to be equivalent to treating the pumping of polaritons as a constant. The reservoir density is constant in time and space and equivalent to the steady-state density of the reservoir, in the case without an external potential (if $V_{\text{eff}}(x) \equiv 0$), n_0, as can be seen in panel (d). Panels (a-c) show the same patterns that were seen in the plots of the main-text, in which we simulated the time-evolution of the BEC via Eq. (3), instead of Eq. (4).

We now turn our attention to the case of a spatially depending pump $\mathcal{P} \to \mathcal{P}\,(1 - \cos kx)$. The results for this simulation are shown in Fig. 2.

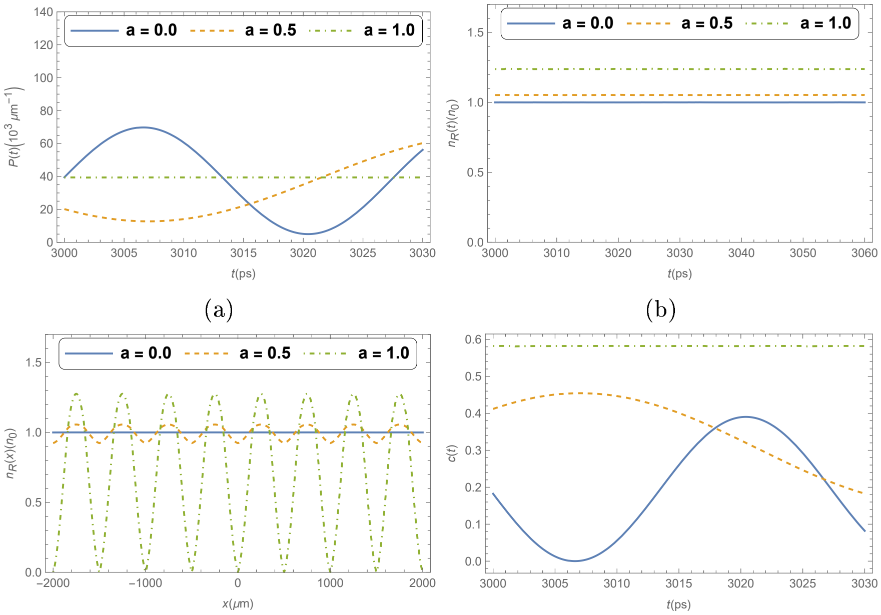

(a) (b) (c) (d)

Figure 2: Numerical results for the time evolution of a Polariton BEC coupled to a polariton reservoir. In this simulation, we considered a spatially dependent pump of polaritons to the reservoir $\mathcal{P}\,(1 - a\cos kx)$. In all panels, the solid blue line corresponds to the case of constant $\mathcal{P}$ (a=0), the dashed yellow line corresponds to the case in which a = 0.5, and the dot-dashed green line corresponds to the case in which $a = 1.0$ (a) Polariton BEC density profile as a function of time at an arbitrary position for different values of the parameter a. (b) Time evolution of the polariton reservoir density at an arbitrary position in the strip. (c) Density profile of the polariton reservoir at long t. (d) Two point correlator $c(t)$, as defined in Eq. (2), for the order parameter $\rho(x,t) = \dfrac{P(x,t) - P_0}{P_0}$.

An analysis of the panels of Fig. 2 shows great similarities with the spatially varying gain/loss analysis done in the main text. We see the polariton BEC reaching a steady state when $a = 1.0$, but we can see evidences of time crystallization in the $a < 1$ regime. Just like in the case in which $\mathcal{P}$ is constant, we still see the reservoir density reaching a steady state. That being said, when $a > 0$, this steady state is no longer constant throughout the strip. The new steady state shows clear similarities with the density profile of the BEC, having peaks of density in exactly the same positions as those of the BEC. One difference being that the BEC peaks pulse in time, while the reservoir density profile is constant in time.

---

[1] Wilckzek, F. Quantum Time Crystals, Phys. Rev. Lett. **109**, 160401 (2012).
[2] Watanabe, H. & Oshikawa, M. Absence of Quantum Time Crystals, Phys. Rev. Lett. **114**, 251603 (2015).
[3] Thies, M. Semiclassical time crystal in the chiral Gross- Neveu model, *preprint*, arXiv:1411.4236 (2014)
[4] Nalitov, A. V. *et al.*, Optically trapped polariton condensate as a semiclassical time crystal Phys. Rev. A **99**, 033830 (2019).
[5] Wouters, M. and Carusotto, I. Excitations in a nonequilibrium Bose-Einstein condensate of exciton polaritons. Phys. Rev. Lett., **99**, 140402 (2007).
[6] Gargoubi, H. Guillet, T. Jaziri, S. Balti, J., & Guizal, B. Polariton condensation threshold investigation through the numerical resolution of the generalized Gross-Pitaevskii equation. Phys. Rev. E, **94**, 043310 (2016).
